# Versatile Role of Rab27a in Glioma: Effects on Release of Extracellular Vesicles, Cell Viability, and Tumor Progression

**DOI:** 10.3389/fmolb.2020.554649

**Published:** 2020-11-12

**Authors:** Thomas S. van Solinge, Erik R. Abels, Lieke L. van de Haar, Killian S. Hanlon, Sybren L. N. Maas, Rosalie Schnoor, Jeroen de Vrij, Xandra O. Breakefield, Marike L. D. Broekman

**Affiliations:** ^1^Department of Neurology and Radiology, Massachusetts General Hospital, Harvard Medical School, Boston, MA, United States; ^2^NeuroDiscovery Center, Harvard Medical School, Boston, MA, United States; ^3^Department of Neurosurgery, Leiden University Medical Center, Leiden, Netherlands; ^4^Department of Neurobiology, Harvard Medical School, Boston, MA, United States; ^5^Molecular Neurogenetics Unit, Department of Neurology, Massachusetts General Hospital, Charlestown, MA, United States; ^6^Department of Neurosurgery, UMC Utrecht Brain Center, Utrecht University, Utrecht, Netherlands; ^7^Department of Pathology, University Medical Center Utrecht, Utrecht University, Utrecht, Netherlands; ^8^Department of Neurosurgery, Brain Tumor Center, Erasmus Medical Center, Rotterdam, Netherlands; ^9^Department of Neurosurgery, Haaglanden Medical Center, The Hague, Netherlands

**Keywords:** glioma, glioblastoma, extracellular vesicles, exosomes, tumor microenvironment, Rab27a

## Abstract

**Introduction:** Glioma cells exert influence over the tumor-microenvironment in part through the release of extracellular vesicles (EVs), membrane-enclosed structures containing proteins, lipids, and RNAs. In this study, we evaluated the function of Ras-associated protein 27a (Rab27a) in glioma and evaluated the feasibility of assessing its role in EV release in glioma cells *in vitro* and *in vivo*.

**Methods:** Rab27a was knocked down via a short hairpin RNA (shRNA) stably expressed in mouse glioma cell line GL261, with a scrambled shRNA as control. EVs were isolated by ultracentrifugation and quantified with Nanoparticle Tracking Analysis (NTA) and Tunable Resistive Pulse Sensing (TRPS). CellTiter-Glo viability assays and cytokine arrays were used to evaluate the impact of Rab27a knockdown. GL261.shRab27a cells and GL261.shControl were implanted into the left striatum of eight mice to assess tumor growth and changes in the tumor microenvironment.

**Results:** Knockdown of Rab27a in GL261 glioma cells decreased the release of small EVs isolated at 100,000 × *g in vitro* (*p* = 0.005), but not the release of larger EVs, isolated at 10,000 × *g*. GL261.shRab27a cells were less viable compared to the scramble control *in vitro* (*p* < 0.005). A significant increase in CCL2 expression in shRab27a GL261 cells was also observed (*p* < 0.001). However, *in vivo* there was no difference in tumor growth or overall survival between the two groups, while shRab27a tumors showed lower proliferation at the tumor borders. Decreased infiltration of IBA1 positive macrophages and microglia, but not FoxP3 positive regulatory T cells was observed.

**Conclusion:** Rab27a plays an important role in the release of small EVs from glioma cells, and also in their viability and expression of CCL2 *in vitro.* As interference in Rab27a expression influences glioma cell viability and expression profiles, future studies should be cautious in using the knockdown of Rab27a as a means of studying the role of small EVs in glioma growth.

## Introduction

Glioblastoma (GB; WHO grade IV glioma) and lower grade gliomas are known to manipulate and exploit the tumor-microenvironment in the brain (Broekman et al., 2018). One way in which the tumor interacts with the microenvironment is via the secretion of extracellular vesicles (EVs). EVs are membrane-enclosed structures that bud from the cell membrane (microvesicles or ectosomes), or are released by fusion of multi-vesicular endosomes (MVEs) with the plasma membrane (so-called exosomes, or “small EVs”) (Maas et al., 2017). EVs contain an assortment of proteins, lipids, and RNAs, roughly representing the cytosol of the producer cell (Abels and Breakefield, 2016). These EVs are secreted into the extracellular space and can be transferred to adjacent and, to a lesser extent, distant cells (Van Niel et al., 2018).

In GB, tumor-derived EVs have been shown to support progression via several mechanisms. First of all, these EVs help create an immune suppressed environment in the brain, compounding to the already low intrinsic immunogenicity of GB (Abels et al., 2019a). Glioma-derived EVs have been shown to modify the phenotype of invading monocytes, macrophages, and microglia, inducing tumor-supportive transcriptomic and phenotypic changes in these cells (De Vrij et al., 2015; Van Der Vos et al., 2016; Abels et al., 2019b). In addition to the effects on the innate immune system, local as well as peripheral T-cell activation is hindered by the expression of PD-L1 on GB-derived EVs (Harshyne et al., 2016; Ricklefs et al., 2018). Furthermore, GB-derived EVs have been shown to modify astrocytes toward a tumor-supportive phenotype (Oushy et al., 2018; Hallal et al., 2019). Glioma cells also use EVs to communicate amongst themselves: oncogenic drivers can be exchanged, most well-described for the Epidermal Growth Factor Receptor (EGFRvIII) pathway (Al-Nedawi et al., 2008). In general, proliferation and invasion is enhanced by GB EVs *in vitro* and *in vivo*, although the exact mechanisms are not well understood (Skog et al., 2008; Pinet et al., 2016). Finally, GB EVs contribute to the typical aberrant neo-vascularization and increased vascular permeability seen in GB, by containing various proteolytic enzymes (Giusti et al., 2016), chemokine receptors (Treps et al., 2016), pro-angiogenic growth factors (Treps et al., 2017), and pro-angiogenic miRNAs (Lucero et al., 2020).

As the detrimental effects of tumor-derived EVs are being revealed, efforts are being made to decrease the release of EVs in various cancers (Bobrie et al., 2012; Datta et al., 2017; Nishida-Aoki et al., 2017). In *in vitro* models, knockdown of Ras-associated protein 27a (Rab27a), implicated as a driver in exosome release, is a well-known method of decreasing release of small EVs. Rab27a controls the fusion of MVEs to the plasma membrane, initiating the release of small EVs (i.e., exosomes), into the extracellular space (Ostrowski et al., 2010; Bobrie et al., 2012). Knockdown of Rab27a has been shown to decrease the shedding of small EVs in fibrosarcoma (Sung et al., 2015), melanoma (Peinado et al., 2012), prostate (Webber et al., 2015), and breast cancer (Bobrie et al., 2012) cell lines.

In this study, we sought to assess the role of Rab27a in EV release in glioma. We show that knockdown of Rab27a decreased release of small exosomes, as detected by Nanoparticle Tracking Analysis (NTA) and Tunable Resistive Pulse Sensing (TRPS). However, knockdown of Rab27a also had various off-target downstream effects, hindering cell growth *in vitro* and changing cytokine release. Knockdown of Rab27a did not affect release of larger EVs. *In vivo*, no clear differences in tumor growth were seen in a mouse syngeneic brain tumor model, while changes in immune cell infiltration were observed. These effects raise caution in interpreting effects of altering Rab27a expression in studying the effects of exosome release in gliomas. Furthermore, it encourages further research into the functions of Rab27a in glioma and the role of other, larger, EVs in tumor-to-microenvironment communication.

## Results

### Rab27a Knockdown Decreases EV Release

**FIGURE 1:**
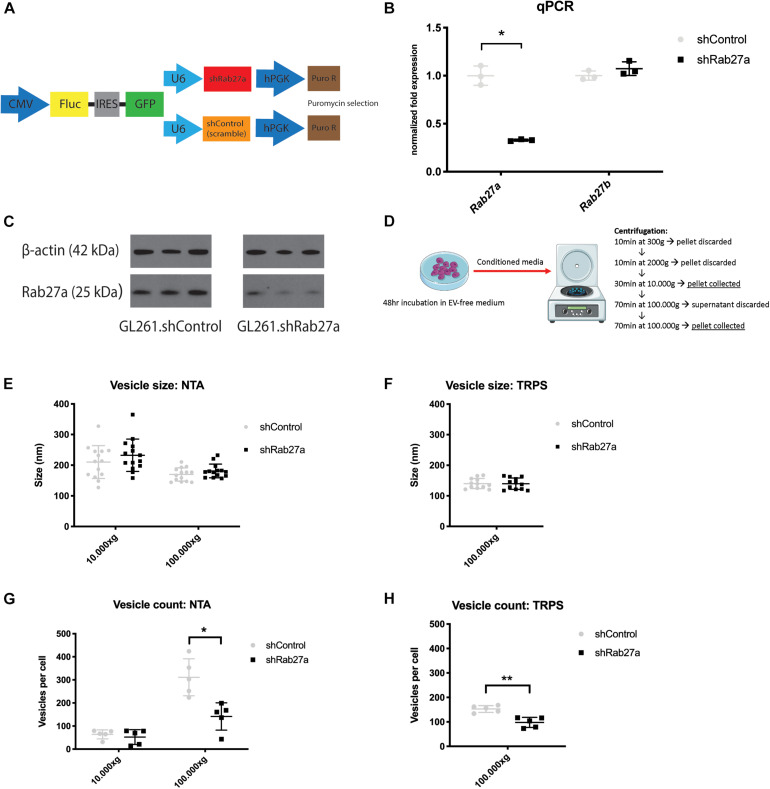
Effects of Rab27a knockdown on *in vitro* EV release. **(A)** Constructs used to transduce GL261 glioma cell line to create GL261.Fluc.GFP.shRab27a/shControl. CMV, cytomegalovirus promoter; Fluc, firefly luciferase; IRES, internal ribosome entry site; GFP, green fluorescent protein; shRab27a, short hairpin Ras-associated protein 27a; shControl, short hairpin control; hPGK, human phosphoglycerate kinase promoter. **(B)** mRNA expression of Rab27a and Rab27b, shControl versus shRab27a, as determined by qPCR. Fold expression normalized to GAPDH mRNA, three replicates per condition. Mean with standard deviation (SD), independent student *t*-test: *p < 0.03. **(C)** Western blot for Rab27a and β-actin in shControl versus shRab27a cells, in triplicate. kDa, kilo dalton. **(D)** Vesicle isolation protocol via ultracentrifugation. **(E)** Vesicle size in nm as determined by nanoparticle tracking analysis (NTA) in 100,000 and 10,000 × *g* fractions. Mean with SD. **(F)** Vesicle size in nm as determined by tunable resistive pulse sensing (TRPS) in 100,000 × g fraction. Mean with SD. **(G)** Vesicle count per cell as determined by NTA in 100,000 and 10,000 × *g* fractions. Mean with SD, independent *t*-test: *p < 0.05. **(H)** Vesicle count per cell as determined by TRPS in 100,000 × *g* fraction. Mean with SD, independent student-*t* test: **p < 0.01.

To knockdown Rab27a, we stably transduce GL261 cells with a lentiviral vector bearing an expression cassette encoding a short hairpin RNA (shRNA) targeting Rab27a mRNA. Our GL261 cell line had previously been transduced to express Firefly luciferase and GFP (GL261.Fluc.GFP) (Figure 1A). Three shRNAs were tried, but only one showed consistent viability of the cells (Supplementary Table 1). As a control, GL261.Fluc.GFP were transduced with a non-targeting shRNA (shControl). Significant Rab27a knockdown by over 70% was confirmed via RT-qPCR, while expression of Rab27b was not affected (independent *t*-test: *p* = 0.03 and *p* = 0.3, respectively; Figure 1B). Protein levels of Rab27a were diminished after knockdown, as confirmed by western blot (Figure 1C). EVs were isolated from culture media using a differential ultracentrifugation protocol (Figure 1D) (Théry et al., 2018). We opted to isolate and evaluate EVs collected at 10,000 and 100,000 × *g*. The size of the EVs was analyzed with two different characterization methods. NTA detects and quantifies vesicles by illuminating the particles in suspension with a laser and capturing the light scatter under a microscope. A camera tracks the particles and using the Stoke–Einstein equation the individual size of the particles is calculated (Dragovic et al., 2011). NTA showed that the EVs in the 10,000 × *g* fraction were significantly larger compared to those in the 100,000 × *g* fraction (independent *t*-test: *p* = 0.0003), and that there was no difference in size between vesicles isolated from GL261.shRab27a and GL261.shControl cells (Figure 1E). EVs isolated at 100,000 × *g* were additionally profiled using TRPS. TRPS quantifies particles by passing them through a nanopore located in a non-conductive polyurethane membrane separating two fluid cells. A flow of ions is induced across the membrane. As a particle crosses this field it alters the flow of ions, creating a brief resistive pulse from which the size of the particle can be deduced (Roberts et al., 2010). TRPS confirmed the similar size of small EVs between the shRab27a and the shControl cells isolated at 100,000 × *g* (Figure 1F). In the 100,000 × *g* fraction, the number of EVs released per cell was significantly reduced in shRab27a cells compared to the control (independent *t*-test, *p* = 0.038) (Figure 1G). This decrease was not observed in the 10,000 × *g* fraction. Again, TRPS confirmed the decrease in number of small EVs isolated at 100,000 × *g* in shRab27a cells (independent *t*-test, *p* < 0.01) (Figure 1H). These results show that downregulating the expression of Rab27a reduced the number of small EVs released per cell.

### Knockdown of Rab27a Interferes With Cell Viability *in vitro*

**FIGURE 2:**
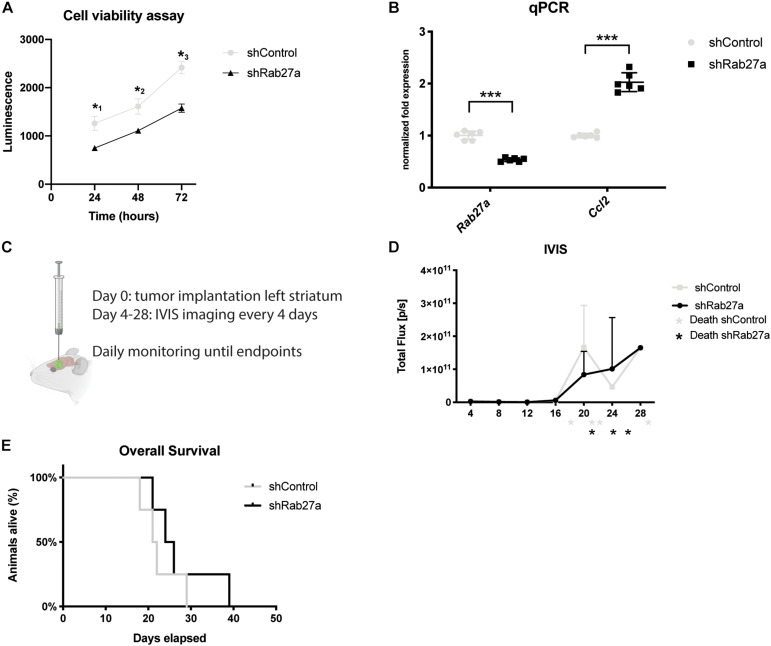
Effects of Rab27 on tumor growth. **(A)** Cell viability as measured by Cell Titer Glow. shControl.GL261 versus shRab27a.GL261. Three replicates per condition at three consecutive time points. *P*-values: *^1^0.048, *^2^0.045, *^3^0.014. **(B)** Fold expression of Rab27a and CCL2 via qPCR, normalized to GAPDH. Mean with SD, independent *t*-test: ***p < 0.001. **(C)** Schematic of tumor injection and time schedule. **(D)** Expression of firefly luciferase (Fluc) as an indicator of tumor size was determined by *in vivo* imaging system (IVIS) in total flux per second from day 4 until day 28. Four mice per group. * indicates death or euthanasia of mice in respective group. **(E)** Overall survival. End-point was defined as death, or euthanasia due to >20% decrease in weight, presence of lethargy, or debilitating neurological symptoms. Four mice per group. Log-rank test: *p* = 0.35.

Since the docking of MVEs to the plasma membrane is part of normal cell physiology, we also investigated if interference in these pathways would alter cell viability. Cell viability was assessed using the Cell Titer Glo assay which quantifies the number of viable cells by metabolic activity based on ATP levels (Zakowick et al., 2008). We detected a 40% decrease in cell viability 24 h after plating the shRab27a cells compared to the scrambled shControl (independent *t*-test: *p* = 0.048), increasing to a 50% difference after 72 h (independent *t*-test: *p* < 0.014) (Figure 2A). As dysregulation of cytokine release plays an important part in glioma pathogenesis, we investigated downstream effects of Rab27a knockdown by evaluating the cytokine release in the media after 72 h (Supplementary Figures 1A–C) (Zhu et al., 2012). The array showed a major increase in expression of CC-chemokine ligand 2 (CCL-2), and minor upregulation of other cytokines such as TIMP-1 and CCL5. The stark increase in CCL2 peaked our interest, as it is essential for regulating the regulatory T cell response and stimulating immunosuppression in GB (Chang et al., 2016), and can promote tumor invasiveness (Zhang et al., 2012), while inhibition of its main receptor CCR2 reduces myeloid infiltration and improves the effect of checkpoint inhibition in glioma (Flores-Toro et al., 2020). RT-qPCR showed a nearly twofold upregulation of CCL2 mRNA in shRab27a glioma cells (independent *t*-test, *p* < 0.0001) (Figure 2B). This shows that in addition to reducing secretion of small EVs, the downregulation of Rab27a alters cell viability and cytokine release from GL261 cells.

### Assessment of Rab27a Knockdown *in vivo*

To further assess the impact of Rab27a knockdown on GL261 glioma cells, we investigated the growth dynamics of the two cell lines, stably transduced with lentivirus packaged with an expression cassette for firefly luciferase (Fluc), *in vivo*. To this end, we injected GL261 cells in the striatum of eight C57BL/6 mice: four with shRab27a cells, and four with shControl cells (scrambled shRNA) (Figure 2C). Fluc was used to monitor the growth of the tumor with *in vivo* bioluminescence imaging over time (Supplementary Figure 1D). Endpoints where determined as either a >20% decrease in body weight, presence of lethargy, or debilitating neurological symptoms.

The tumor-derived Fluc signal decreased until day 12, after which tumors in both groups showed exponential growth (Figure 2D). This growth pattern is typical for implantations with GL261 (Abdelwahab et al., 2011). There was no difference in average size of tumors (as estimated by Fluc signal and post-mortem gross pathology) or rate of tumor growth. After 40 days, all mice had reached their end-points. Mice implanted with shRab27a GL261 tumors had a median survival of 24 days, compared to 21 days in the control group (Figure 2E, log-rank test: *p* = 0.35).

**FIGURE 3:**
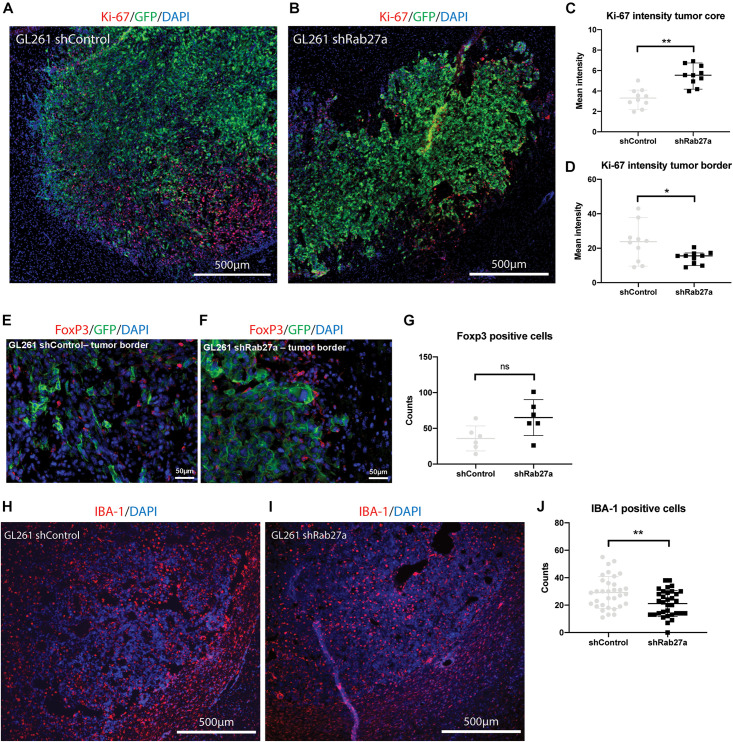
*In vivo* characterization of tumors. All tumors were acquired after death from the tumor, or euthanasia due to >20% decrease in weight, presence of lethargy, or debilitating neurological symptoms. **(A)** Representative immunohistochemical staining of GL261.shcontrol tumor and surrounding tissue. Red: Ki-67, green: GFP, blue: DAPI. **(B)** GL261.shRab27a tumor. Red: Ki-67, green: GFP, blue: DAPI. **(C)** Ki-67 expression as measured by mean intensity per field of view in the tumor core. Mean and standard error. Independent *t*-test: **p = 0.001. **(D)** Ki-67 expression as measured by mean intensity per field of view in the tumor border. Mean and standard error. Independent *t*-test: *p = 0.03. **(E)** Representative immunohistochemical staining of GL261.shcontrol tumor border. Red: FoxP3, green: GFP, blue: DAPI. **(F)** GL261.shRab27a tumor border. Red: FoxP3, green: GFP, blue: DAPI. **(G)** Number of FoxP3 positive cells per field of view. Mean and standard error. NS, not significant. **(H)** Representative immunohistochemical staining of a GL261.shControl tumor and surrounding tissue. Red: IBA-1, blue: DAPI. **(I)** GL261.shRab27a tumor. Red: IBA-1, blue: DAPI. **(J)** IBA-1 positive cells as counted per field of view in ImageJ. Mean and standard error. Independent *t*-test: **p = 0.006.

As we observed lower viability and cell growth for GL261.shRab27a *in vitro*, we stained tumors for Ki-67, a cell proliferation marker (Scholzen and Gerdes, 2000), after the mice had reached their end-points (Figures 3A,B). Measuring mean intensity in 10 randomly selected areas, we observed a higher intensity for Ki-67 in the tumor core of shRab27a (Figure 3C, independent *t*-test, *p* = 0.001), while intensity for Ki-67 was higher in the tumor border of shControl tumors (Figure 3D, independent *t*-test, *p* = 0.03). As CCL2 has been implicated in recruitment of regulatory T cells (Chang et al., 2016), we manually counted the number of FoxP3, a marker for regulatory T cells (Li et al., 2015), positive cells in shControl and shRab27a tumors. We found a slight increase in the number of regulatory T cells in shRab27a tumors, but this difference was not significant (Figures 3E–G).

Finally, we investigated changes in infiltration of microglia and macrophages via staining with IBA-1 (Figures 3H–J) (Ahmed et al., 2007). We performed an automated count of the number of IBA-1 positive cells using ImageJ, randomly selecting 11 areas for three different mice per condition (33 areas where counted in total per condition) (example shown in Supplementary Figure 1E). Median number of IBA-1 positive cells per area was 22 in the shRab27a group versus 29 in the shControl group (Figure 3J, independent *t*-test, *p* = 0.006). This finding was confirmed by a second researcher, blinded to the previous outcome and nature of the tumors (560 μm by 290 μm, *n* = 15 per condition. Median IBA-1 positive cells in the shRab27a group: 131 versus 185 in shControl group, *p* = 0.03. *Data not shown*).

## Discussion

In this study, we confirmed the role of Rab27a in the release of small EVs in glioma *in vitro* and showed that knockdown of Rab27a resulted in reduced cell viability and increased expression of CCL2. *In vivo*, growth of tumors and overall survival of mice did not differ after implantation in the striatum, although glioma cells showed higher proliferation at the tumor border in the shControl tumors. Tumor infiltration of IBA-1 positive cells was significantly decreased after Rab27a knockdown, while infiltration of FoxP3 positive regulatory T cells showed an upward trend.

The mechanisms by which EVs are released are multifold. In broad terms, large EVs (microvesicles) are generally released by budding off of the plasma membrane or nanotubules, while small EVs (exosomes) are released through fusion of multivesicular endosomes with the plasma membrane (Abels and Breakefield, 2016). While the importance of EVs in cancer progression is becoming increasingly apparent (Sato and Weaver, 2018), our understanding of the underlying molecular mechanisms contributing to specific cargo packaging, vesicle release, and modes of communication remains limited. Rab27a facilitates docking of MVEs to the plasma membrane, one of the final steps in exosome release (Ostrowski et al., 2010; Van Niel et al., 2018). Indeed, here we show that knockdown of Rab27a decreased release of small EVs in glioma cells in culture, as previously described for other cancer cell lines (Bobrie et al., 2012; Peinado et al., 2012; Sung et al., 2015; Webber et al., 2015). Although decreasing the release of EVs in general by cancer cells might restrict tumor progression, it is important to note that EVs are heterogeneous (Mathieu et al., 2019). EVs produced by cancer cells contain specific proteins, RNAs, and other molecules related to tumor growth and metastasis, and there is increasing awareness that some EVs are packaged and released via cancer specific mechanisms (Clancy et al., 2015; Sato and Weaver, 2018). At least a subset of EVs released from cancer cells have unique properties as compared to those from normal cells. Guo et al. (2019) showed that small EVs isolated by ultracentrifugation from melanoma cells with high Rab27a expression contained much higher concentrations of proteins related to cancer cell invasion and metastasis compared to EVs isolated from human melanoma cells with loss of Rab27a expression. Furthermore, decreased Rab27a expression in these cells decreased invasion and cell motility *in vitro* and *in vivo. In vitro* this could be partly rescued by adding small EVs isolated from Rab27a expressing cells, while adding small EVs isolated from Rab27a knockout cells did not have this effect. This suggests that Rab27a has a role in regulating the types of EVs released and their contents, enhancing their tumor stimulatory effects in cancer cells. Currently, we could not evaluate the changes in EV release *in vivo* in our model. We assume that the effects on vesicle release observed *in vitro* mimic the effects *in vivo*, but *in vivo* circumstances, such as hypoxia, cell stress, and necrosis could be more dominant regulators of EV release (Caruso and Poon, 2018; Shao et al., 2018). Methods to evaluate EV release from tumors *in vivo* (Van Der Vos et al., 2016), which are not yet quantitative, could provide valuable insight into these processes. Moreover, aside from the traditional methods of EV release, cancer cells appear to have additional pathways for shedding EVs. Oncosomes are larger vesicles, specifically found in cancers, which bleb from the cell membrane (Minciacchi et al., 2015a,b). In prostate cancer cells, oncosomes can be separated from small EVs via discontinuous centrifugation gradient (Minciacchi et al., 2015b). Treatment of these cells with oncosomes led to changes in aspartate transaminase metabolism, while small EVs did not have this effect (Minciacchi et al., 2015b). Apoptotic bodies, previously considered as cell debris, have been implicated in various cancer processes, ranging from immune modulation to stimulation of tumor growth (Lynch et al., 2017; Caruso and Poon, 2018). More nuance is needed in describing these various particles, as they are hard to distinguish from one another and clear consensus on defining features is currently lacking (Théry et al., 2018; Mathieu et al., 2019). It could very well be that *in vivo*, additional cell stress, caused by hypoxic conditions and necrosis, stimulates the shedding of apoptotic bodies and/or oncosomes, compensating for the signals lost due to Rab27a knockdown.

Previous work has shown that Rab27a plays an important role in cell viability and proliferation in a range of cancers. Reduced proliferation and invasion has been described in melanoma after knockdown of Rab27a (Akavia et al., 2010; Guo et al., 2019), while in breast cancer cell growth was decreased (Bobrie et al., 2012). In bladder cancer, knockdown reduced invasion and high expression was corelated with poor prognosis (Ostenfeld et al., 2014). This relation between high expression of Rab27a and poor prognosis has been found in many cancers, such as lung cancer (Koh and Song, 2019), melanoma (Akavia et al., 2010; Guo et al., 2019), and hepatocellular carcinoma (Dong et al., 2012). In glioma, there is an increasing body of research noting the importance of Rab27a in viability and aggressiveness of these tumors. In patients, increased Rab27a expression has been linked to poorer survival and malignant progression of GB (Wang et al., 2014). Recently, short hairpin mediated knockdown of Rab27a in 73C glioma cells showed decreased growth *in vitro* and *in vivo* in a mouse model (Gao et al., 2020). siRNA mediated knockdown of Rab27a in C6 and U251 glioma cell lines has also resulted in a decrease in cell proliferation and invasion (Liu et al., 2012). Conversely, overexpression of Rab27a has been shown to increase cell viability, proliferation, and invasion in U251 cells (Wu et al., 2013). We observed a significant impact of Rab27a on cell viability: shRab27a GL261 cells showed decreased viability *in vitro* compared to the GL261 cells transduced with a shControl. As the transduction itself did not interfere with the phenotype or viability, with shControl cells showing similar growth and viability compared to GL261 wild-type (*data not shown*), it seems unlikely that this was the root of the decrease in viability. *In vivo*, we observed a slight increase in expression of proliferation marker Ki-67 in the core of shRab27a tumors, while at the border Ki-67 intensity was weaker. As the tumor core in fast growing tumors tends to be hypoxic and necrotic, it is possible that the proliferation here decreases at the later stages of tumor growth and that a faster growing tumor leads to less viable cells in the tumor core. In patients, GB with significant necrosis on MRI has been related to poorer outcomes and more aggressive tumors (Hammoud et al., 1996). The infiltrating tumor cells at the tumor border showed higher expression of Ki-67 in the control, suggesting that shControl tumors have a slightly more aggressive outward growth, corresponding to our data *in vitro*.

For our study, we focused on the effects of Rab27a in the syngeneic GL261 mouse glioma model. We chose this model because it has been extensively used *in vivo* and thoroughly characterized (Szatmári et al., 2006). Furthermore, implantation can be done in immunocompetent mice, allowing for analysis of changes in the (immune) tumor microenvironment. More studies are needed to address the role of Rab27a in glioma in different cell lines and *in vivo* models. Interestingly, Gao et al. (2020) showed a stark difference in tumor growth after Rab27a knockdown in 73C cells, noting a 30-fold decrease *in vivo*. This highlights the fact that different glioma cell lines might respond differently to knockdown of Rab27a, with some cell lines more affected than others. The exact mechanisms underlying Rab27a expression and cell viability in glioma and cancer in general remain to be further understood.

We found that knockdown of Rab27a led to a marked increase in expression of CCL2, a cytokine vital in mediating macrophage, micoglia, and regulatory T cell infiltration in glioma (Chang et al., 2016). We observed a decrease in IBA-1 positive cells in shRab27a GL261 tumors, while infiltration of FoxP3 regulatory T cells was slightly increased, albeit not significant. In mice, blocking of CCL2 has been shown to increase the antiangiogenic effects of bevacizumab (Cho et al., 2019). Knockdown of CCL2 via siRNA inhibited cell proliferation and angiogenesis in the glioma cell line U251 *in vitro* (Lu et al., 2017) while overexpression in a U87 glioma cell line increased invasiveness, dependent on presence of CCR2-expressing microglia (Zhang et al., 2012). The decrease in infiltration of IBA-1 cells in shRab27a tumors is therefore surprising. EVs play a vital role in tumor to cell communication (Van Der Vos et al., 2016; Broekman et al., 2018) and it was recently shown that PD-L1 released via EVs is essential for suppressing T cell activation in glioma (Poggio et al., 2019). It could be possible that the decrease in EV release negates any immune-stimulatory effects that the increase of CCL2 release has on the tumor microenvironment. In our study, we did not analyze the presence of PD-L1 in the 10.000 or 100.000 × *g* fraction, so we were not able to address if EV mediated PD-L1 release was diminished. Another caveat is that we were not able to quantify the levels of CCL2 *in vivo*, data that can be important to elucidate whether the increase *in vitro* pertains *in vivo.* These experiments should be done in future studies to further our knowledge of the interaction between Rab27a and CCL2. The underlying mechanisms are currently unclear and remain to be further understood.

Recent *in vitro* work has shown that GB-derived vesicles can modify the phenotype of monocytic cells, inducing expression of EGFR and secretion of IL-6, increasing phagocytosis, and reducing levels of c-Myc (De Vrij et al., 2015; Van Der Vos et al., 2016). In our model, we saw that macrophage and microglial infiltration was decreased after knockdown of Rab27a. However, given the previously discussed impact of Rab27a knockdown on CCL2 expression, this model may not be able to pin-point the exact mechanisms to study glioma–macrophage interactions via EVs. We also acknowledge that various factors can interfere with accurate cell count, such as areas of necrosis, and further studies need to be done to confirm this effect.

The problem of off-target effects of Rab27a knockdown has been noted before. Peinado et al. (2012) found a decrease in the secretion of various growth factors, such as placental growth factor 2 (PlGF-2) and platelet-derived growth factor A chain homodimer (PDGF-AA) after knockdown of Rab27a in melanoma cells, and Bobrie et al. (2012) observed a decrease in the release of matrix metalloproteinase 9 after knockdown of Rab27a in HeLa cells. This further warrants caution in ascribing observed effects to a decrease in the release of small EVs if achieved via knockdown of Rab27a.

In summary, we showed that knockdown of Rab27a decreased the release of small EVs, hampered viability of GL261 cells, and increased secretion of the chemokine CCL2 *in vitro*. *In vivo*, tumor growth and overall survival was not affected by knockdown of Rab27a, but lower Ki-67 expression was observed at the tumor border. Infiltration of IBA-1 positive macrophages and microglia was decreased in GL261.shRab27a tumors, while infiltration of FoxP3 regulatory T cells showed an upward trend. The exact relation between Rab27a expression, EV release, cell viability, and cytokine release needs to be further elucidated before Rab27a can be used to study EVs in glioma biology or used with therapeutic intent. New discoveries of ways in which tumor cells release EVs, such as oncosomes and apoptotic bodies, underline the notion that interfering with a single mode of EVs release might be of little therapeutic value in cancer.

## Materials and Methods

### Cell Culture

Cell culture and viral transduction details can be found in the Supplementary Methods.

### Viability Assay

In a 96-well, 10,000 cells were plated per well and incubated overnight at 37°C in 5% CO_2_. CellTiter-Glo^®^ (Promega, Madison, WI, United States) reagents were mixed and added to the wells the after 24/48/72 h. After 2 min of mixing and 10 min of incubation at room temperature, luminescence was recorded on a Synergy HTX platform (BioTek Instruments, Winooski, VT, United States).

### Extracellular Vesicle Isolation

1.5 × 10^6^ cells/dish were plated on 150 mm dishes. After 24 h, the medium was removed, and the cells were incubated for 48 h in 20 ml EV-depleted FBS supplemented medium [RPMI with 5% FBS (EV-depleted) + 1% penicillin/streptomycin + 2 μl/ml puromycin]. FBS was depleted of EVs by 16-h ultracentrifugation at 160,000 × *g*. Conditioned medium was then collected and centrifuged according to the following protocol: 300 × *g* for 10 min, 2000 × *g* for 10 min both at room temperature, then 10,000 × *g* for 30 min at 4°C (saving the pellet) followed by centrifugation of the supernatant at 100,000 × *g* at 4°C for 70 min. The last supernatant was then discarded, the pellet resuspended, and again centrifuged at 100,000 × *g* at 4°C for 70 min. The 10,000 × *g* pellet and the final 100,000 × *g* pellet were isolated for EV analysis. The pellets were diluted 2× in 0.22 μm filtered PBS (1%) and stored at −80°C for RNA analysis. Vesicle quantification and analysis was done directly after isolation.

### Vesicle Quantification

Quantification of vesicles was performed using the NanoSight LM10 NTA (Salisbury, Great Britain) with NTA 3.1 software. Movies were captured for 30 s with settings (camera-level 12); slider shutter 1187 and slider gain 286. For analysis, screen gain was set at 10 and the detection threshold at 3.0. After collection of the media, cells were trypsinized and counted by hand to correct for the number of cells. The 100,000 × *g* fractions were also quantified with TRPS using the qNANO (Izon Science, Cambridge, MA, United States) and Izon Control Suite 3.2 software. The NP200 (A30771) nanopore was used with the following settings: applied stretch: 46.69 mm, applied voltage: 0.46 V, average current: 112 nA, average noise: 9.6 nA, maximum particle count: 500 particles, and maximum duration: 120 s. All samples were measured twice in separate blocks according to the same settings. All samples were calibrated using CPC200 calibration particles (Izon Science) in a 1:1000 dilution in 2 × 0.2 μm filtered PBS.

### Western Blotting

Details can be found in the Supplementary Methods.

### Quantitative Real-Time PCR

Details can be found in the Supplementary Methods.

### Cytokine Array

300,000 cells were seeded in a six-well plate. 500 μl of media was collected after 72 h. Samples were spun for 10 min at 300 × *g* to remove any residual cells. The cytokine array was performed using the Proteome Profiler Array Mouse Cytokine Array Panel A (R&D Systems, Minneapolis, MN, United States), following the manufacturer’s protocol. Quantification was done in ImageJ using the Dot Blot Analyzer for ImageJ by Gilles Carpentier, Universite de Paris, France (Carpentier, 2008).

### Animal Care

C57BL/6 (Charles River Laboratories Wilmington, MA, United States) aged between 8 and 12 weeks were used for all experiments. Animals were housed at the animal facility of the Massachusetts General Hospital under standard laboratory conditions, with free access to water and food. After injection of tumor cells, mice were monitored daily to assess health, appearance and behavior. Mice were weighed three times a week. All experiments were approved under IACUC protocol 2009N000054.

### Intracranial Tumor Cell Injections

C57BL/6 mice were anesthetized using isoflurane and their heads secured in a stereotactic frame. A skin incision was made and Bregma was localized. A burr-hole was made, and 100,000 cells suspended in 2 μl OptiMEM (Thermo-Fisher, Waltham, MA, United States) were injected at the estimated position of the left striatum, 2.0 mm anterior and 0.5 mm lateral from Bregma, 2.5 mm deep from the skull surface. The incision was closed with Hystoacryl glue (Braun) and a single dose of 100 μg/kg buprenorphine (Temgesic, BD Pharmaceutical Ltd., Franklin Lakes, NJ, United States) was administered as an analgesic. The experimental group was injected with GL261.Fluc.GFP.shRab27a cells, the control with GL261.Fluc.GFP.shControl cells. Endpoint was either >20% weight loss or animal distress, at which time mice were sacrificed.

### Bioluminescence Imaging

100 μl D-Luciferin (Thermo-Fischer) (25 mg/ml in saline) was injected intra-peritoneally in isoflurane-anesthetized mice. After 10 min, the mice were imaged using the IVIS (*In Vivo* Imaging System) Spectrum connected to an X GI-8 Anesthesia System (PerkinElmer, Waltham, MA, United States). Bioluminescence was expressed as total flux per second.

### Immunohistochemistry

Mice were perfused transcardial with PBS, followed by 4% paraformaldehyde (PFA) (Electron Microscopy Sciences, Hatfield, PA, United States). Brain were then removed and stored in PFA overnight. After cryoprotection in 30% sucrose for 48 h, samples were frozen in NEG-50 (Thermo-Fischer) at −80°C. Brains were cut on a cryostat at 12 μm thickness. For FoxP3, antigen retrieval was performed for 15 min in boiling sodium citrate, 10 mM pH 6.0 + 0.5% Tween 20. For staining, the sections were permeabilized with 0.5% Triton-X in PBS for 1 h and blocked with 5% Normal Goat Serum (Invitrogen, Carlsbad, CA, United States) for 1 h. Primary staining was done overnight at 4°, secondary for 1 h at room temperature. Primary antibodies: Rabbit polyclonal IBA-1 (019-19741) antibody from Wako Chemicals (Richmond, VA, United States) at 1:400, Ki67 (ab15580, Abcam, Cambridge, United Kingdom) at 1:100, FoxP3 (D608R, Cell Signaling, Danvers, MA, United States) at 1:100. Secondary: Alexa Fluor 647 goat anti-Rabbit (A21245) (Invitrogen) at 1:1000. Images were acquired on the Nikon Eclipse TE 2000-U (Nikon Instruments Inc., Melville, NY, United States) and the BZ-X microscope (Keyence, Itasca, IL, United States). ImageJ 2.0.0v software was used to process the images and create Z-stacks. Analysis was done with NIS-Elements v4.6 software (Nikon Instruments Inc.) and ImageJ v2.0 (National Institutes of Health, United States).

### Cell Counting

Slides were stained for IBA-1 and DAPI and imaged as noted above. The TRITC channel images were exported independently, converted to black and white, and inversed. Eleven frames, measuring 220 μm by 150 μm were selected at random within a section of the tumor. Three different slides from three different mice were used per condition. Using the ITCN plug-in version 1.6 (T. Kuo, J. Byun. Centre for Bio-Image informatics, University of Santa Barbara) IBA1 positive cells were automatically counted. A second, independent investigator blinded to the study performed the analysis in similar fashion, using an area measuring 560 by 390 μm on the same slides as the previous researcher. An example is shown in Supplementary Figure 1D.

For FoxP3, 6 fields were randomly selected and cells were counted manually in Adobe Photoshop (Adobe, San Jose, CA, United States). For Ki-67, pictures were converted to grayscale and 10 fields were randomly selected in the tumor center and tumor border. Counting was done by hand and confirmed by an independent investigator. Mean Gray Value was then calculated in ImageJ.

### Statistical Analysis

Individual statistical tests are noted in Section “Results” and figure legends. Log-rank test was performed with Prism 8 (GraphPad Software, Inc., San Diego, CA, United States). All other analyses were performed in R v3.4.1 (The R Foundation for Statistical Computing). Graphs were made using Prism 8 (GraphPad Software). Independent *t*-tests were performed two-tailed. *P*-values < 0.05 were considered as statistically significant.

### Artwork

Illustrations used in Figure 1D were obtained from Servier Medical Art (Les Laboratories Servier, France). Illustrations used in Figure 2C were created with BioRender.com under the Harvard license. Figures were made in Adobe Illustrator (Adobe, San Jose, CA, United States).

## Data Availability Statement

The raw data supporting the conclusions of this article will be made available by the authors upon request, without undue reservation.

## Ethics Statement

The animal study was reviewed and approved by the Massachusetts General Hospital Animal Committee under IACUC protocol 2009N000054.

## Author Contributions

TS, EA, JV, XB, and MB contributed to conception and design of the study. TS, EA, LH, KH, SM, and RS performed the experiments. TS performed the statistical analysis. TS wrote the first draft of the manuscript. TS, EA, LH, and KH wrote sections of the manuscript. TS and KH made the figures and images. XB and MB supervised the project. All authors contributed to manuscript revision, read, and approved the submitted version.

## Conflict of Interest

The authors declare that the research was conducted in the absence of any commercial or financial relationships that could be construed as a potential conflict of interest.

## Supplementary Material

Supplementary Figure 1 **(A)** Exploratory cytokine array, GL261.shRab27a on the right, GL261.shControl on the left. **(B)** Quantification with the Dot Blot Analyzer in ImageJ. **(C)** Signal intensity of cytokine array as measured with the Dot Blot Analyzer. **(D)** Example of IVIS imaging at 16 days post tumor implantation. **(E)** Example of cell count performed by ITCN-plugin in ImageJ. Image shows shRab27a.GL261 tumor at 10×. Cut-out is shown of the counted area, red dots mark cells counted by the software. In the cut-out, the red dots have been highlighted in Adobe Illustrator for illustrative purposes.

## References

[B1] Abdelwahab M. G. Sankar T. Preul M. C. Scheck A. C. (2011). Intracranial implantation with subsequent 3D in vivo bioluminescent imaging of murine gliomas. *J. Vis. Exp.* 407:e3403. 10.3791/3403 22158303 PMC3308614

[B2] Abels E. R. Breakefield X. O. (2016). Introduction to extracellular vesicles: biogenesis, RNA cargo selection, content, release, and uptake. *Cell Mol. Neurobiol.* 36 301–312. 10.1007/s10571-016-0366-z 27053351 PMC5546313

[B3] Abels E. R. Broekman M. L. D. Breakefield X. O. Maas S. L. N. (2019a). Glioma EVs contribute to immune privilege in the brain. *Trends Cancer* 5 393–396. 10.1016/j.trecan.2019.05.006 31311653 PMC8237701

[B4] Abels E. R. Maas S. L. N. Nieland L. Wei Z. Cheah P. S. Tai E. (2019b). Glioblastoma-associated microglia reprogramming is mediated by functional transfer of extracellular miR-21. *Cell Rep.* 28 3105.e7–3119.e7. 10.1016/j.celrep.2019.08.036 31533034 PMC6817978

[B5] Ahmed Z. Shaw G. Sharma V. P. Yang C. McGowan E. Dickson D. W. (2007). Actin-binding proteins coronin-1a and IBA-1 are effective microglial markers for immunohistochemistry. *J. Histochem. Cytochem.* 55 687–700. 10.1369/jhc.6A7156.2007 17341475

[B6] Akavia U. D. Litvin O. Kim J. Sanchez-Garcia F. Kotliar D. Causton H. C. (2010). An integrated approach to uncover drivers of cancer. *Cell* 143 1005–1017. 10.1016/j.cell.2010.11.013 21129771 PMC3013278

[B7] Al-Nedawi K. Meehan B. Micallef J. Lhotak V. May L. Guha A. (2008). Intercellular transfer of the oncogenic receptor EGFRvIII by microvesicles derived from tumour cells. *Lett. Nat. Cell Biol.* 10 619–624. 10.1038/ncb1725 18425114

[B8] Bobrie A. Krumeich S. Reyal F. Recchi C. Moita L. F. Seabra M. C. (2012). Rab27a supports exosome-dependent and -independent mechanisms that modify the tumor microenvironment and can promote tumor progression. *Cancer Res.* 72 4920–4930. 10.1158/0008-5472.CAN-12-0925 22865453

[B9] Broekman M. L. Maas S. L. N. Abels E. R. Mempel T. R. Krichevsky A. M. Breakefield X. O. (2018). Multidimensional communication in the microenvirons of glioblastoma. *Nat. Rev. Neurol.* 14 482–495. 10.1038/s41582-018-0025-8 29985475 PMC6425928

[B10] Carpentier G. (2008). *Dot Blot Analyzer.* Available online at: http://image.bio.methods.free.fr/dotblot.html (accessed August 10, 2020).

[B11] Caruso S. Poon I. K. H. (2018). Apoptotic cell-derived extracellular vesicles: more than just debris. *Front. Immunol.* 9:1486. 10.3389/fimmu.2018.01486 30002658 PMC6031707

[B12] Chang A. L. Miska J. Wainwright D. A. Dey M. Rivetta C. V. Yu D. (2016). CCL2 produced by the glioma microenvironment is essential for the recruitment of regulatory T Cells and myeloid-derived suppressor cells. *Cancer Res.* 76 5671–5682. 10.1158/0008-5472.CAN-16-0144 27530322 PMC5050119

[B13] Cho H. R. Kumari N. Thi Vu H. Kim H. Park C. K. Choi S. H. (2019). Increased antiangiogenic effect by blocking CCL2-dependent macrophages in a rodent glioblastoma model: correlation study with dynamic susceptibility contrast perfusion MRI. *Sci. Rep.* 9 1–12. 10.1038/s41598-019-47438-4 31366997 PMC6668454

[B14] Clancy J. W. Sedgwick A. Rosse C. Muralidharan-Chari V. Raposo G. Method M. (2015). Regulated delivery of molecular cargo to invasive tumour-derived microvesicles. *Nat. Commun.* 6 1–11. 10.1038/ncomms7919 25897521 PMC4497525

[B15] Datta A. Kim H. Lal M. McGee L. Johnson A. Moustafa A. A. (2017). Manumycin A suppresses exosome biogenesis and secretion via targeted inhibition of Ras/Raf/ERK1/2 signaling and hnRNP H1 in castration-resistant prostate cancer cells. *Cancer Lett.* 408 73–81. 10.1016/j.canlet.2017.08.020 28844715 PMC5628151

[B16] De Vrij J. Niek Maas S. L. Kwappenberg K. M. C. Schnoor R. Kleijn A. Dekker L. (2015). Glioblastoma-derived extracellular vesicles modify the phenotype of monocytic cells. *Int. J. Cancer* 137 1630–1642. 10.1002/ijc.29521 25802036

[B17] Dong W. W. Mou Q. Chen J. Cui J. T. Li W. M. Xiao W. H. (2012). Differential expression of Rab27A/B correlates with clinical outcome in hepatocellular carcinoma. *World J. Gastroenterol.* 18 1806–1813. 10.3748/wjg.v18.i15.1806 22553406 PMC3332295

[B18] Dragovic R. A. Gardiner C. Brooks A. S. Tannetta D. S. Ferguson D. J. P. Hole P. (2011). Sizing and phenotyping of cellular vesicles using nanoparticle tracking analysis. *Nanomed. Nanotechnolo. Biol. Med.* 7 780–788. 10.1016/j.nano.2011.04.003 21601655 PMC3280380

[B19] Flores-Toro J. A. Luo D. Gopinath A. Sarkisian M. R. Campbell J. J. Charo I. F. (2020). CCR2 inhibition reduces tumor myeloid cells and unmasks a checkpoint inhibitor effect to slow progression of resistant murine gliomas. *Proc. Natl. Acad. Sci. U.S.A.* 117 1129–1138. 10.1073/pnas.1910856117 31879345 PMC6969504

[B20] Gao X. Zhang Z. Mashimo T. Shen B. Nyagilo J. Wang H. (2020). Gliomas interact with non-glioma brain cells via extracellular vesicles. *Cell Rep.* 30 2489.e5–2500.e5. 10.1016/j.celrep.2020.01.089 32101730

[B21] Giusti I. Delle Monache S. Di Francesco M. Sanità P. D’Ascenzo S. Gravina G. L. (2016). From glioblastoma to endothelial cells through extracellular vesicles: messages for angiogenesis. *Tumor. Biol.* 37 12743–12753. 10.1007/s13277-016-5165-0 27448307

[B22] Guo D. Lui G. Y. L. Lai S. L. Wilmott J. S. Tikoo S. Jackett L. A. (2019). RAB27A promotes melanoma cell invasion and metastasis via regulation of pro-invasive exosomes. *Int. J. Cancer* 144 3070–3085. 10.1002/ijc.32064 30556600

[B23] Hallal S. Mallawaaratchy D. M. Wei H. Ebrahimkhani S. Stringer B. W. Day B. W. (2019). Extracellular vesicles released by glioblastoma cells stimulate normal astrocytes to acquire a tumor-supportive phenotype Via p53 and MYC signaling pathways. *Mol. Neurobiol.* 56 4566–4581. 10.1007/s12035-018-1385-1 30353492 PMC6505517

[B24] Hammoud M. A. Sawaya R. Shi W. Thall P. E. Leeds N. E. (1996). Prognostic significance of preoperative MRI scans in glioblastoma multiforme. *J. Neurooncol.* 27 65–73. 10.1007/bf00146086 8699228

[B25] Harshyne L. A. Nasca B. J. Kenyon L. C. Andrews D. W. Hooper D. C. (2016). Serum exosomes and cytokines promote a T-helper cell type 2 environment in the peripheral blood of glioblastoma patients. *Neuro Oncol.* 18 206–215. 10.1093/neuonc/nov107 26180083 PMC4724173

[B26] Koh H. M. Song D. H. (2019). Prognostic role of Rab27A and Rab27B expression in patients with non-small cell lung carcinoma. *Thorac. Cancer* 10 143–149. 10.1111/1759-7714.12919 30480360 PMC6360262

[B27] Li Z. Li D. Tsun A. Li B. (2015). FOXP3+ regulatory T cells and their functional regulation. *Cell Mol. Immunol.* 12 558–565. 10.1038/cmi.2015.10 25683611 PMC4579651

[B28] Liu Y. Zhou Y. Zhu K. (2012). Inhibition of glioma cell lysosome exocytosis inhibits glioma invasion. *PLoS One* 7:e0045910. 10.1371/journal.pone.0045910 23029308 PMC3461042

[B29] Lu B. Zhou Y. Su Z. Yan A. Ding P. (2017). Effect of CCL2 siRNA on proliferation and apoptosis in the U251 human glioma cell line. *Mol. Med. Rep.* 16 3387–3394. 10.3892/mmr.2017.6995 28714025

[B30] Lucero R. Zappulli V. Sammarco A. Murillo O. D. Cheah P. S. Srinivasan S. (2020). Glioma-derived miRNA-containing extracellular vesicles induce angiogenesis by reprogramming brain endothelial cells. *Cell Rep.* 30 2065.e4–2074.e4. 32075753 10.1016/j.celrep.2020.01.073 PMC7148092

[B31] Lynch C. Panagopoulou M. Gregory C. D. (2017). Extracellular vesicles arising from apoptotic cells in tumors: roles in cancer pathogenesis and potential clinical applications. *Front. Immunol.* 8:1174. 10.3389/fimmu.2017.01174 29018443 PMC5614926

[B32] Maas S. L. N. Breakefield X. O. Weaver A. M. (2017). Extracellular vesicles: unique intercellular delivery vehicles. *Trends Cell Biol.* 27 172–188. 10.1016/j.tcb.2016.11.003 27979573 PMC5318253

[B33] Mathieu M. Martin-Jaular L. Lavieu G. Théry C. (2019). Specificities of secretion and uptake of exosomes and other extracellular vesicles for cell-to-cell communication. *Nat. Cell Biol.* 21 9–17. 10.1038/s41556-018-0250-9 30602770

[B34] Minciacchi V. R. Freeman M. R. Di Vizio D. (2015a). Extracellular vesicles in cancer: exosomes, microvesicles and the emerging role of large oncosomes. *Semin. Cell Dev. Biol.* 40 41–51. 10.1016/j.semcdb.2015.02.010 25721812 PMC4747631

[B35] Minciacchi V. R. You S. Spinelli C. Morley S. Zandian M. Aspuria P.-J. (2015b). Large oncosomes contain distinct protein cargo and represent a separate functional class of tumor-derived extracellular vesicles. *Oncotarget* 6 11327–11341. 10.18632/oncotarget.3598 25857301 PMC4484459

[B36] Nishida-Aoki N. Tominaga N. Takeshita F. Sonoda H. Yoshioka Y. Ochiya T. (2017). Disruption of circulating extracellular vesicles as a novel therapeutic strategy against cancer metastasis. *Mol. Ther.* 25 181–191. 10.1016/j.ymthe.2016.10.009 28129113 PMC5363297

[B37] Ostenfeld M. S. Jeppesen D. K. Laurberg J. R. Boysen A. T. Bramsen J. B. Primdal-Bengtson B. (2014). Cellular disposal of miR23b by RAB27-dependent exosome release is linked to acquisition of metastatic properties. *Cancer Res.* 74 5758–5771. 10.1158/0008-5472.CAN-13-3512 25261234

[B38] Ostrowski M. Carmo N. B. Krumeich S. Fanget I. Raposo G. Savina A. (2010). Rab27a and Rab27b control different steps of the exosome secretion pathway. *Nat. Cell Biol.* 12 19–30. 10.1038/ncb2000 19966785

[B39] Oushy S. Hellwinkel J. E. Wang M. Nguyen G. J. Gunaydin D. Harland T. A. (2018). Glioblastoma multiforme-derived extracellular vesicles drive normal astrocytes towards a tumour-enhancing phenotype. *Philos. Trans. R. Soc. Lond. B. Biol. Sci.* 373:20160477. 10.1098/rstb.2016.0477 29158308 PMC5717433

[B40] Peinado H. Alečković M. Lavotshkin S. Matei I. Costa-Silva B. Moreno-Bueno G. (2012). Melanoma exosomes educate bone marrow progenitor cells toward a pro-metastatic phenotype through MET. *Nat. Med.* 18 883–891. 10.1038/nm.2753 22635005 PMC3645291

[B41] Pinet S. Bessette B. Vedrenne N. Lacroix A. Richard L. Jauberteau M.-O. (2016). TrkB-containing exosomes promote the transfer of glioblastoma aggressiveness to YKL-40-inactivated glioblastoma cells. *Oncotarget* 7 50349–50364. 10.18632/oncotarget.10387 27385098 PMC5226587

[B42] Poggio M. Hu T. Pai C. C. Chu B. Belair C. D. Chang A. (2019). Suppression of Exosomal PD-L1 induces systemic anti-tumor immunity and memory. *Cell* 177 414.e13–427.e13. 10.1016/j.cell.2019.02.016 30951669 PMC6499401

[B43] Ricklefs F. L. Alayo Q. Krenzlin H. Mahmoud A. B. Speranza M. C. Nakashima H. (2018). Immune evasion mediated by PD-L1 on glioblastoma-derived extracellular vesicles. *Sci. Adv.* 4:eaar2766. 10.1126/sciadv.aar2766 29532035 PMC5842038

[B44] Roberts G. S. Kozak D. Anderson W. Broom M. F. Vogel R. Trau M. (2010). Tunable Nano/Micropores for particle detection and discrimination: scanning ion occlusion spectroscopy. *Small* 6 2653–2658. 10.1002/smll.201001129 20979105

[B45] Sato S. Weaver A. M. (2018). Extracellular vesicles: important collaborators in cancer progression. *Essays Biochem.* 62 149–163. 10.1042/EBC20170080 29666212 PMC6377252

[B46] Scholzen T. Gerdes J. (2000). The Ki-67 protein: from the known and the unknown. *J. Cell Physiol.* 182 311–322. 10.1002/(SICI)1097-4652(200003)182:3<311::AID-JCP1<3.0.CO;2-9 10653597

[B47] Shao C. Yang F. Miao S. Liu W. Wang C. Shu Y. (2018). Role of hypoxia-induced exosomes in tumor biology. *Mol. Cancer* 17 1–8. 10.1186/s12943-018-0869-y 30098600 PMC6087002

[B48] Skog J. Würdinger T. van Rijn S. Meijer D. H. Gainche L. Sena-Esteves M. (2008). Glioblastoma microvesicles transport RNA and proteins that promote tumour growth and provide diagnostic biomarkers. *Nat. Cell Biol.* 10 1470–1476. 10.1038/ncb1800 19011622 PMC3423894

[B49] Sung B. H. Ketova T. Hoshino D. Zijlstra A. Weaver A. M. (2015). Directional cell movement through tissues is controlled by exosome secretion. *Nat. Commun.* 6:7164. 10.1038/ncomms8164 25968605 PMC4435734

[B50] Szatmári T. Lumniczky K. Désaknai S. Trajcevski S. Hídvégi E. J. Hamada H. (2006). Detailed characterization of the mouse glioma 261 tumor model for experimental glioblastoma therapy. *Cancer Sci.* 97 546–553. 10.1111/j.1349-7006.2006.00208.x 16734735 PMC11159227

[B51] Théry C. Witwer K. W. Aikawa E. Alcaraz M. J. Anderson J. D. Andriantsitohaina R. (2018). Minimal information for studies of extracellular vesicles 2018 (MISEV2018): a position statement of the International Society for Extracellular Vesicles and update of the MISEV2014 guidelines. *J. Extracell Vesicles* 7:1535750. 10.1080/20013078.2018.1535750 30637094 PMC6322352

[B52] Treps L. Edmond S. Harford-Wright E. Galan-Moya E. M. Schmitt A. Azzi S. (2016). Extracellular vesicle-transported Semaphorin3A promotes vascular permeability in glioblastoma. *Oncogene* 35 2615–2623. 10.1038/onc.2015.317 26364614

[B53] Treps L. Perret R. Edmond S. Ricard D. Gavard J. (2017). Glioblastoma stem-like cells secrete the pro-angiogenic VEGF-A factor in extracellular vesicles. *J. Extracell Vesicles* 6:1359479. 10.1080/20013078.2017.1359479 28815003 PMC5549846

[B54] Van Der Vos K. E. Abels E. R. Zhang X. Lai C. Carrizosa E. Oakley D. (2016). Directly visualized glioblastoma-derived extracellular vesicles transfer RNA to microglia/macrophages in the brain. *Neuro Oncol.* 18 58–69. 10.1093/neuonc/nov244 26433199 PMC4677420

[B55] Van Niel G. D’Angelo G. Raposo G. (2018). Shedding light on the cell biology of extracellular vesicles. *Nat. Rev. Mol. Cell Biol.* 19 213–228. 10.1038/nrm.2017.125 29339798

[B56] Wang H. Zhao Y. Zhang C. Li M. Jiang C. Li Y. (2014). Rab27a was identified as a prognostic biomaker by mRNA profiling, correlated with malignant progression and subtype preference in gliomas. *PLoS One* 9:e0089782. 10.1371/journal.pone.0089782 24587032 PMC3935941

[B57] Webber J. Spary L. Sanders A. Chowdhury R. Jiang W. Steadman R. (2015). Differentiation of tumour-promoting stromal myofibroblasts by cancer exosomes. *Oncogene* 34 319–331. 10.1038/onc.2013.560 24441045

[B58] Wu X. Hu A. Zhang M. Chen Z. (2013). Effects of Rab27a on proliferation, invasion, and anti-apoptosis in human glioma cell. *Tumor. Biol.* 34 2195–2203. 10.1007/s13277-013-0756-5 23553027

[B59] Zakowick H. Schagat T. Yoder D. Niles A. L. (2008). Measuring cell health and viability sequentially by same-well multiplexing using the GloMax^®^ -Multi Detection System. *Promega Notes* 99 25–28.

[B60] Zhang J. Sarkar S. Cua R. Zhou Y. Hader W. Wee Yong V. (2012). A dialog between glioma and microglia that promotes tumor invasiveness through the CCL2/CCR2/interleukin-6 axis. *Carcinogenesis* 33 312–319. 10.1093/carcin/bgr289 22159219

[B61] Zhu V. F. Yang J. LeBrun D. G. Li M. (2012). Understanding the role of cytokines in glioblastoma multiforme pathogenesis. *Cancer Lett.* 316 139–150. 10.1016/j.canlet.2011.11.001 22075379

