# Supplementary material for: Versatile Role of Rab27a in Glioma: Effects on Release of Extracellular Vesicles, Cell Viability, and Tumor Progression
Source: Front Mol Biosci. 2020 Nov 12;7:554649. doi: 10.3389/fmolb.2020.554649 (PMC7691322; doi:10.3389/fmolb.2020.554649)
Supplement: Supplementary file 1 [file Data_Sheet_1.PDF]

# Versatile Role Of Rab27a In Glioma: Effects On Release Of Extracellular Vesicles, Cell Viability And Tumor Progression

*Thomas S. van Solinge[1,2], Erik R. Abels[1], Lieke L. van de Haar[1], Killian S. Hanlon[3,4], Sybren L.N. Maas[5,6], Rosalie Schnoor[5], Jeroen de Vrij[7], Xandra O. Breakefield[1], Marike L.D. Broekman[1,2,8].*

# Supplementary

## Supplementary methods:

*Cell culture*

Cells were cultured in Roswell Park Memorial Institute (RPMI) medium 1640 with L-Glutamine (Corning, Corning, NY, USA) supplemented with 10% Fetal Bovine Serum (FBS) (Sigma-Aldrich, Saint Louis, MO, USA) and 1% penicillin/streptomycin (P/S) (Invitrogen, Carlsbad, CA, USA). GL261.Fluc.GFP.shRab27a and GL261.Fluc.GFP.shControl cells were cultured under an additional puromycin pressure of 2µg/mL. Cells were incubated at 37°C and 5% $CO_2$.

*Viral transduction*

The CSCW2.Fluc.IRES.GFP plasmid, kindly provided by Dr. Miguel Sena-Esteves (Neurology, University of Massachusetts Medical School), was packaged in a lentiviral vector[1]. The internal ribosome entry site (IRES) element enables simultaneous transcription of Fluc and GFP. 250,000 mouse glioma cells (NCI GL261 0507814) were plated in a 6-well plate and incubated for 24 hours. Cells were then transduced using 10µl of the concentrated lentivirus in RPMI 1640 with L-Glutamine (Corning) supplemented with 10% FBS (Sigma-Aldrich) and 1% P/S (Invitrogen) and 8µg/ml polybrene. The media was replaced with standard growth media after 24 hours. Cells were then sorted for high GFP signal via flow cytometry (Massachusetts General Hospital Flow Cytometry Core, Boston, MA, USA). The sorted cells were then again transduced in a similar fashion with the MISSION© shRNA rab27a lentiviral Plasmid Vector pLKO.1-puro (TRCN0000100576, clone ID: NM_023635.2-502s1d1) and MISSION© Non-Target Control Vector SHC002 Lentiviral Plasmid Vector pLKO.1-puro (Sigma-Aldrich) packaged in a lentiviral vector. Cells were grown under 2µg/ml puromycin to select for transduced cells.

*Western Blotting*

Cells were lysed using RIPA buffer (Abcam, Cambridge, MA, USA) with a cocktail of Protease Inhibitors (Roche, Mannheim, Germany) and centrifuged for 10 minutes at 12,000 x g. The protein concentration was quantified using the DC Protein Assay (Bio-Rad Laboratories Inc., Hercules, CA, USA). For blotting, 10μg of protein was suspended in SDS-buffer (Boston Bio Products, Boston, MA, USA) and denatured for 10 minutes at 70°C. Proteins were separated using 4-12% NuPAGE Bis-Tris Gel (ThermoFisher Scientific) and transferred to a nitrocellulose membrane (Bio-Rad). After blocking with Tris-Buffered Saline (TBS) with 0.05% Tween20 (TBS-T) and 5% milk for 1 hour, the membrane was incubated with the primary antibody in TBS-T with 3% milk overnight at 4°C. The membrane was washed with TBS-T and incubated with the secondary antibody for 1 hour at room temperature. Followed by another washing, SuperSignal West Femto (Thermo Scientific) was added and the signal captured on Autoradiography Film (Genesee Scientific, San Diego, CA, USA). Primary antibodies: rabbit monoclonal to Rab27a (D7Z9Q) 1:100 (Cell Signalling Technology, Danvers, MA, USA), rabbit anti-β-actin (1801-100) 1:500 (Abcam). Secondary antibodies: ECL anti-rabbit IgG Horse-Radish peroxidase 1:1000 (GE Healthcare).

*RNA extraction*

RNA was extracted using the miRNeasy kit (Qiagen, Hilden, Germany) according to the manufacture's protocol including the optional DNase digestion. RNA quality and concentration was evaluated with the NanoDrop Spectophotometer ND-1000 (ThermoFisher Scientific).

*Quantative real-time PCR*

cDNA was prepared using SuperScript VILO cDNA Synthesis Kit (ThermoFisher Scientific). Quantitative real time PCR (qPCR) was performed according to the Power SYBR Green PCR kit (Applied Biosystems, Foster City, CA, USA) using 1 ng cDNA input on the 7500 Fast Real-Time PCR system (Applied Biosystems). The cycling conditions used were 50°C for 2 min, 90°C for 10 minutes, and 40 cycles of 95°C for 15 seconds and 60°C for 1 minute. PCR amplification was performed in triplicate and normalized to GAPDH expression. Used primers are: mmu-Rab27a-FW: TCGGATGGAGATTACGATTACCT, mmu-Rab27a-RV: TTTTCCCTGAAATCAATGCCCA, mmu-GAPDH-FW: AGGTCGGTGTGAACGGATTTG, mmu-GAPDH-RV: TGTAGACCATGTAGTTGAGGTCA,

mmu-Rab27b-FW: TAGACTTTCGGGAAAAACGTGTG, mmu-Rab27b-RV: AGAAGCTCTGTTGACTGGTGA, mmu-CCL2-FW: TTAAAAACCTGGATCGGAACCAA,

mmu-CCL2-RV: GCATTAGCTTCAGATTTACGGGT.

## Supplementary references


1. Sena-Esteves M, Tebbets JC, Steffens S, Crombleholme T, Flake AW. Optimized large-scale production of high titer lentivirus vector pseudotypes. J Virol Methods 2004;122(2):131–9.

| **Supplementary Table 1** | |
|---|---|
| **shRNA** | **Result** |
| TRCN0000100576, clone: NM_023635.2-502s1d1 | Viable cells |
| TRCN0000100578, clone: NM_023635.2-252s1c1 | Very slow growing cells, signs of cell stress |
| TRCN0000100575, clone: NM_023635.2-1870s1c1 | Cells not viable |
