# Supplementary figures and images for: Versatile Role of Rab27a in Glioma: Effects on Release of Extracellular Vesicles, Cell Viability, and Tumor Progression
Source: Front Mol Biosci. 2020 Nov 12;7:554649. doi: 10.3389/fmolb.2020.554649 (PMC7691322; doi:10.3389/fmolb.2020.554649)

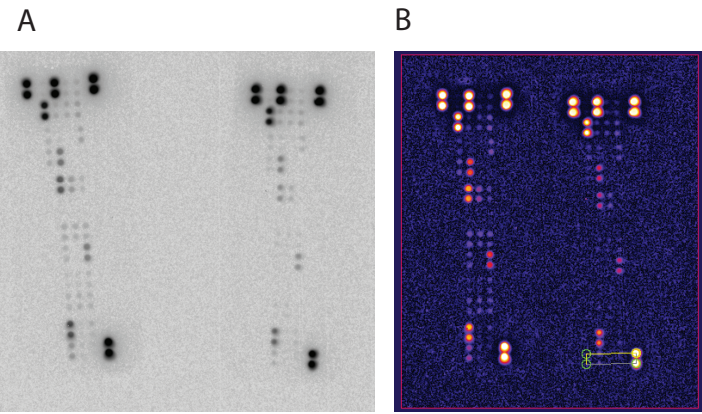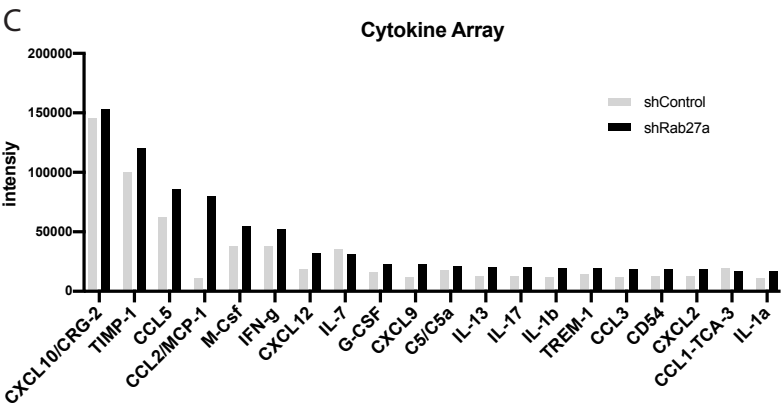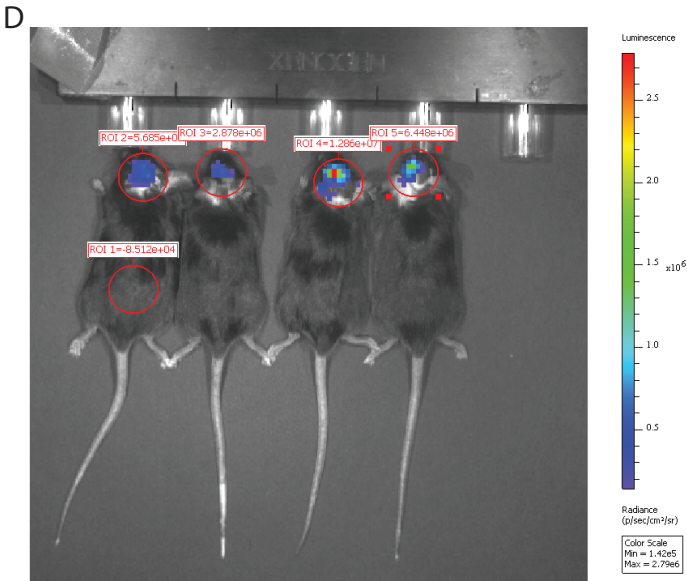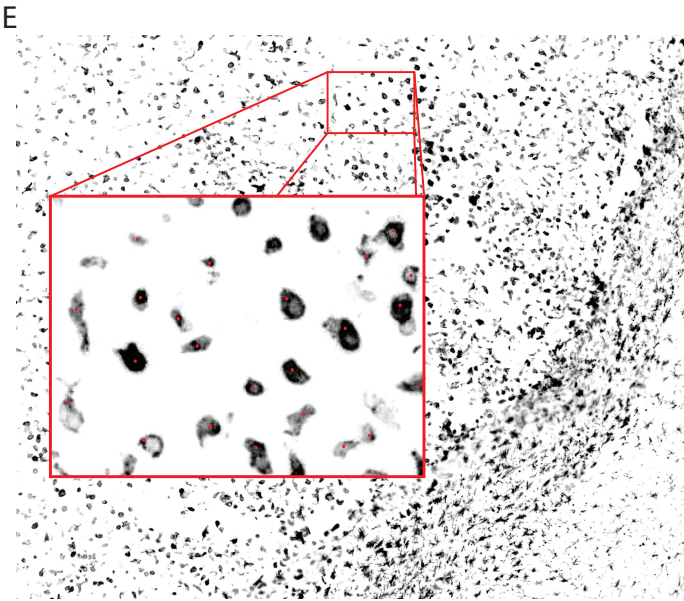

Supplement: Supplementary Figure 1 — (A) Exploratory cytokine array, GL261.shRab27a on the right, GL261.shControl on the left. (B) Quantification with the Dot Blot Analyzer in ImageJ. (C) Signal intensity of cytokine array as measured with the Dot Blot Analyzer. (D) Example of IVIS imaging at 16 days post tumor implantation. (E) Example of cell count performed by ITCN-plugin in ImageJ. Image shows shRab27a.GL261 tumor at 10×. Cut-out is shown of the counted area, red dots mark cells counted by the software. In the cut-out, the red dots have been highlighted in Adobe Illustrator for illustrative purposes. [file Image_1.PDF]
